# *Gtsf1l* and *Gtsf2* Are Specifically Expressed in Gonocytes and Spermatids but Are Not Essential for Spermatogenesis

**DOI:** 10.1371/journal.pone.0150390

**Published:** 2016-03-01

**Authors:** Noriaki Takemoto, Takuji Yoshimura, Satsuki Miyazaki, Fumi Tashiro, Jun-ichi Miyazaki

**Affiliations:** 1 Division of Stem Cell Regulation Research, Osaka University Graduate School of Medicine, 2–2 Yamadaoka, Suita, Osaka, 565–0871, Japan; 2 Laboratory of Reproductive Engineering, Institute of Experimental Animal Sciences, Osaka University Medical School, 2–2 Yamadaoka, Suita, Osaka, 565–0871, Japan; Zhejiang University, CHINA

## Abstract

The unknown protein family 0224 (UPF0224) includes three members that are expressed in germ-line cells in mice: *Gtsf1*, *Gtsf1l*, and *BC048502* (*Gtsf2*). These genes produce proteins with two repeats of the CHHC Zn-finger domain, a predicted RNA-binding motif, in the N terminus. We previously reported that *Gtsf1* is essential for spermatogenesis and retrotransposon suppression. In this study, we investigated the expression patterns and functions of *Gtsf1l* and *Gtsf2*. Interestingly, *Gtsf1l* and *Gtsf2* were found to be sequentially but not simultaneously expressed in gonocytes and spermatids. Pull-down experiments showed that both GTSF1L and GTSF2 can interact with PIWI-protein complexes. Nevertheless, knocking out *Gtsf1*, *Gtsf2*, or both did not cause defects in spermatogenesis or retrotransposon suppression in mice.

## Introduction

In mammals, male reproduction is maintained by successive generations of sperm in the testes. Spermatogenesis involves mitosis, meiosis, and spermiogenesis [1]. Mitosis self-renews the spermatogonia, whose progeny differentiate into primary spermatocytes [2]. Meiosis generates haploid round spermatids and halves the number of chromosomes in the primary spermatocytes. Spermiogenesis, by which the haploid spermatids are processed to mature sperm, occurs in a progressive, organized manner within the seminiferous tubule, and tubule cross-sections reveal spermatogenic cells at 12 distinct stages (I-XII).

Intact spermatogenesis requires a group of genes that silence retrotransposons. Three mouse PIWI proteins, MIWI, MILI, and MIWI2, are reported to silence retrotransposons via PIWI-interacting small RNAs (piRNAs) [3–5]. MIWI, which interacts with TDRD6, is essential for spermiogenesis [6]. MILI interacts with TDRD1 for secondary piRNA biogenesis [7,8,9]. MIWI2 interacts with TDRD9 to silence retrotransposons prior to transcription [10].

We previously used *in silico* screening to identify mouse genes that are specifically expressed in germ-line cells [11], and reported that one of these genes, *Gtsf1/Cue110*, is involved in spermatogenesis and retrotransposon suppression in murine testes [12]. We recently confirmed that *Gtsf1* is also involved in piRNA biogenesis (unpublished). Here, we focused on *Gtsf1l* and on *BC048502*, which we designated *Gtsf2*. We previously showed by RT-PCR analysis that these genes, which belong to the mouse unknown protein family 0224 (UPF0224), are expressed in the testis [11]. However, the expression and function of *Gtsf1l* and *Gtsf2* in spermatogenesis is unclear. In this study, we examined the *Gtsf1l* and *Gtsf2* expression patterns in detail and described the phenotypes of mice lacking *Gtsf1l*, *Gtsf2*, or both.

## Materials and Methods

### Animals

All experiments involving animals were carried out in accordance with institutional guidelines under the protocols (No. 21–089 and No. 26–066), which were approved by the Animal Care and Use Committee of the Osaka University Graduate School of Medicine. Mice were euthanized with an intraperitoneal injection of pentobarbital sodium at 180 mg/kg body weight.

### RT-PCR

Total RNA was extracted from embryonic and postnatal testes at various ages using the acid guanidinium thiocyanate-phenol-chloroform method. Total RNA was pretreated with RNase-free DNase I, and then 1 μg of RNA was reverse-transcribed for 30 min at 42°C in a total volume of 40 μl, using an Oligo(dT) primer and ReverTraAce reverse transcriptase (Toyobo, Osaka, Japan), according to the manufacturer’s instructions. The resulting cDNA was PCR-amplified with gene-specific primers using 20–30 cycles of 98°C for 15 sec, 55–65°C for 30 sec, and 72°C for 60 sec, followed by an extension for 10 min at 72°C. The amplified products were separated by electrophoresis on a 2% agarose gel and detected by ethidium-bromide staining. *Gtsf1l* cDNA was amplified as a 297-bp product using the primer pair 5’-ATGAGAGGAGGGGACCCAGGAGAAAC-3’ and 5’-AAGTGTCCTGCTGCCCAAAGTGTACG-3’. *Gtsf2* cDNA was amplified as a 320-bp product using the primer pair 5’-CCAACTGTATCAACAGGACTGCAGT-3’ and 5’-GGCAATGTCTCCATCAGTTTTTCTGC-3’. As an internal control, *Gapdh* cDNA was amplified as a 983-bp product using the primer pair 5’-CATGTAGGCCATGAGGTCCACCAC-3’ and 5’-TGAAGGTCGGTGTGAACGGATTTGGC-3’.

### Cell culture and transfection

To obtain full-length *Gtsf2* cDNA, the cDNA from mouse testis was amplified by PCR, inserted into a pCAG-IRES-puro vector [13] to produce pGTSF2, and confirmed by sequencing. The monkey kidney-cell line BMT-10 was cultured in Dulbecco’s Modified Eagle’s Medium NP-40 (Sigma-Aldrich, St. Louis, MO) supplemented with 10% fetal bovine serum (BioWhittaker, Walkersville, MD) at 37°C. Cells were transfected with pGTSF2 using Lipofectamine^TM^ 2000 (Invitrogen, Carlsbad, CA).

### Antibodies against GTSF1L and GTSF2

**Fig 1:**
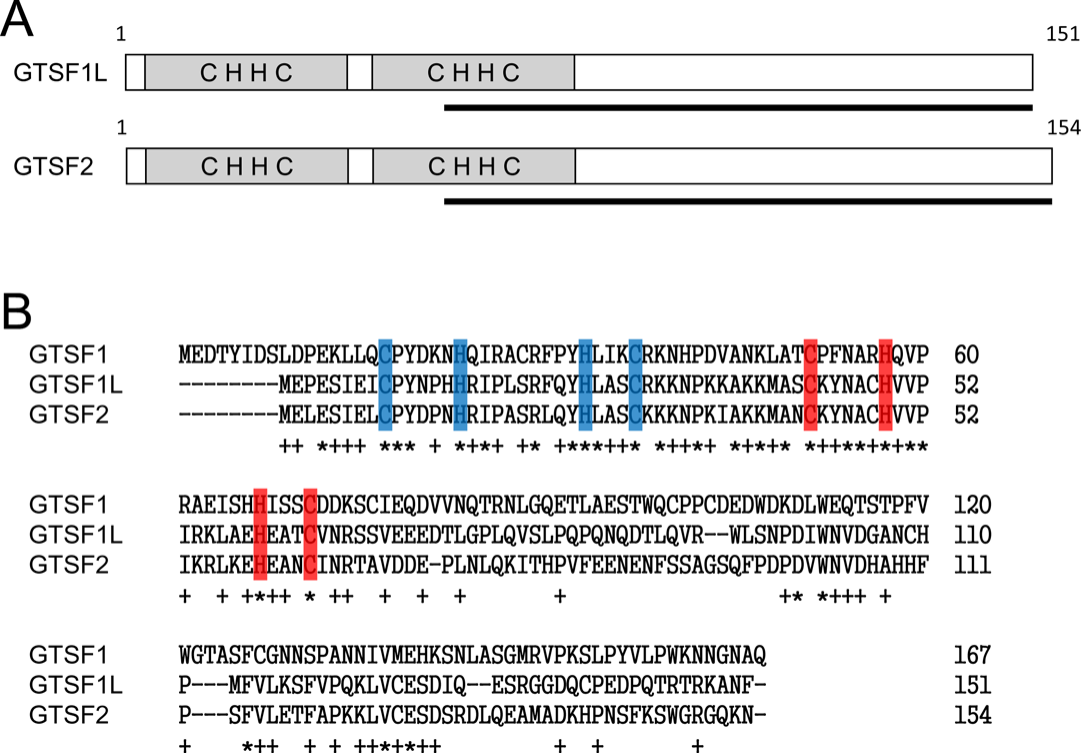
GTSF1L and GTSF2 have two N-terminal CHHC-type Zn-finger domains. (A) Domain architecture of mouse UPF0224-family proteins. The N-terminal region has two CHHC Zn-finger domains. The black lines indicate the regions used to raise rabbit antibodies against GTSF1L and GTSF2. (B) Alignment of the amino-acid residues of the mouse UPF0224-family proteins using CLUSTAL-X. The first and second CHHC Zn-finger domains are indicated in blue and red, respectively. Asterisks indicate amino-acid residues that are conserved among GTSF1, GTSF1L, and GTSF2. Pluses indicate amino-acid residues that are conserved between GTSF1L and GTSF2.

Antibodies against GTSF1L and GTSF2 were generated as described previously [10]. Briefly, cDNA fragments encoding the C-terminal amino-acid residues 53–151 and 53–154 of GTSF1L and GTSF2, respectively (**Fig 1A**), were cloned into the pGEX-6P-3 vector (Amersham, Arlington Heights, IL). The resulting vectors were transferred into *E*. *coli* BL21 cells to produce glutathione-S-transferase (GST)-dN-GTSF1L and GST-dN-GTSF2 fusion proteins, which were purified with Glutathione Sepharose 4 Fast Flow (GE Healthcare, Uppsala, Sweden) and used to immunize rabbits. The antiserum was immunoaffinity-purified. Antibody specificities against GTSF1L and GTSF2 were verified by western blotting (**S3B Fig**) and immunofluorescence analysis (**S3C–S3F Fig**). The antibodies against GTSF1L and GTSF2 did not cross-react with GTSF2 and GTSF1L, respectively.

### Western blotting

Testes were homogenized in TNE buffer [10 mM Tris-HCl, pH 7.5, 1 mM EDTA, 150 mM NaCl, 0.1% NP-40, and Protease Inhibitor Cocktail III (Sigma-Aldrich, St. Louis, MO)]. BMT-10 cells transfected with pGTSF2 were lysed directly using TNE buffer. Each lysate was mixed with a half-volume of 3x SDS Sample Buffer (New England Biolabs, Beverly, MA) and 1/30-volume of 1 M DTT, and heated at 99°C for 5 min. A total of 10 μg protein was loaded per lane, separated on a 15% SDS-polyacrylamide gel, and then transferred onto an Immobilon-P membrane (Millipore, New Bedford, MA). The membranes were blocked with 3% skim milk in TTBS (10 mM Tris-HCl, pH 7.6, 137 mM NaCl, and 0.1% Tween 20) for 1 h at room temperature, incubated overnight at 4°C with anti-GTSF1L (1:2500) or anti-GTSF2 (1:1000) in blocking solution, washed three times in TTBS, and incubated with horseradish peroxidase-conjugated goat anti-rabbit Ig (1:2000; Dako, Kyoto, Japan) in blocking solution for 1 h at room temperature. The membranes were again washed three times in TTBS, and signals were detected with the ECL Western Blotting Detection Kit (GE Healthcare).

### Immunofluorescence

Testes were fixed with 4% paraformaldehyde in PBS overnight at 4°C, washed in PBS, placed in 10% sucrose for 1 h, and incubated in 20% sucrose for 1 h. The tissues were embedded in O.C.T. compound (Sakura Finetek, Tokyo, Japan), cryosectioned at 10 μm, and immunostained with anti-GTSF1L (1:1000), anti-GTSF2 (1:1000), anti-L1ORF1p (1:5000; gifted by Dr. Alex Bortvin), and anti-IAP GAG (1:500) [14]. Next, the samples were washed in PBS and incubated with the secondary antibodies Alexa Fluor 488 or 594 goat anti-rabbit IgG (Molecular Probes, Eugene, OR). After being washed in PBS, the samples were counterstained with 4', 6-diamidino-2-phenylindole (DAPI) for 2 min.

### *Gtsf1l*- and *Gtsf2*-null mice

To generate a *Gtsf1l*-null mouse, we used the CRISPR/Cas9 system and a double-nicking strategy [15,16]. Two plasmids containing the expression cassettes for hCas9 (D10A) and sgRNA were constructed by ligating two annealed single-stranded oligo DNAs including the CRISPR target sequence (**S1A Fig**) into the BbsI site of the pX335 vector (www.addgene.org/CRISPR). Cryopreserved pronuclear-stage BDF1 eggs (CLEA Japan, Tokyo, Japan) were thawed and cultured for 10 min in EmbryoMax KSOM Medium with 1/2 Amino Acids (Cat. #MR-106-D; Merck-Millipore, Darmstadt, Germany). The two pX335 plasmids (5 ng/μl each) were microinjected into the pronuclei of each egg, the eggs were cultured in the same medium overnight, and two-celled embryos were transferred into the oviducts of pseudopregnant female ICR mice. To genotype founder mice, genomic fragments containing the CRISPR target sequence were PCR-amplified using PrimeSTAR MAX (Takara, Shiga, Japan) and sequenced.

To generate a *Gtsf2*-null mouse, we applied a conventional gene-targeting strategy using the embryonic stem (ES) cell line KY1.1 [17], established from F_1_ blastocysts generated by mating C57BL/6J and 129S6/SvEvTac mice. The ES cells were cultured on gelatin-coated dishes in Knockout DMEM (Life Technologies, Carlsbad, CA) supplemented with 15% FBS, 1x Nonessential Amino Acids (Life Technologies), 1x sodium pyruvate (Life Technologies), 0.1 mM 2-mercaptoethanol (Wako, Osaka, Japan), 10^3^ units/ml ESGRO (Merck-Millipore), 3 μM CT99021 (Axon, Groningen, Netherlands), and 1 μM PD0325901 (Axon). Homologous genomic fragments for the targeting vector were PCR-amplified from the genomic DNA of C57BL/6N mice using PrimeSTAR MAX (Takara). The resultant targeting vector (**S2A Fig**) was introduced into the ES cells by electroporation. The genomic DNAs from puromycin-resistant colonies were prepared, and homologous recombinants were confirmed by long genomic PCR for the 5’ and 3’ homologous sequences and by Southern blotting with a 5’ probe (**S2B Fig**) and a probe for the Pgk-puro cassette. The targeted ES clones were injected into blastocysts collected from superovulated C57BL/6N female mice, and the blastocysts were transferred into the uterus of pseudopregnant female ICR mice (SLC Japan) to obtain chimeric mice. Male chimeric mice were mated with female C57BL/6N mice, resulting in germ-line transmission of the floxed *Gtsf2* allele. The mice were genotyped by PCR using the primers 5’-CCTAAACTTCTTGCATTGACACAGTAC-3’ and 5’-CAAGGTTCCATCCTTGTCAAAGGCTGTGAC-3’. *Gtsf2*^+/-^ mice were obtained by crossing *Gtsf2*^flox/+^ and CAG-Cre transgenic mice [18]. *Gtsf2*^-/-^ mice were obtained by intercrossing *Gtsf2*^+/-^ mice.

### GST pull-down assay

cDNA fragments encoding the amino-acid residues of GTSF1L [full length: 1–151, C-terminal deletion (dC): 1–111, N-terminal region (ZnF): 1–67, N-terminal deletion (dZnF): 68–151, or central region (CR): 92–111] or GTSF2 (full length: 1–154, dC: 1–112, ZnF: 1–67, dZnF:68–154, or CR: 91–112) were inserted into the pGEX-6P-3 (Invitrogen) cloning site, and the resulting plasmids were transferred into *E*. *coli* BL21 cells. GST or GST-fusion proteins were produced by exposing BL21 cells to 1 mM isopropyl β-D-1-thiogalactopyranoside for 3 h at 30°C. The cells were suspended in lysis buffer (20 mM Tris-HCl, pH 7.5, 1 mM EDTA, 200 mM NaCl, 14 mM 2-mercaproethanol, 1 mM PMSF, and 50 mg/l lysozyme), shaken for 30 min on ice, and sonicated. After centrifugation at 6000 x g for 15 min, the supernatants were mixed with Glutathione Sepharose 4B (GE Healthcare), washed with lysis buffer, and incubated for 2.5 h with gentle rocking to allow GST-fusion proteins to bind to the beads. The beads were washed twice with lysis buffer containing 0.5% Triton X-100, and twice with binding buffer (20 mM Tris-HCl pH 7.5, 150 mM NaCl, and 0.1% NP40). Testis lysates, untreated or treated with 40 U/ml RNase A, were added to the cleared GST-fusion-protein-bound beads, incubated for 2 h at 4°C with gentle rocking, and triple-washed with the binding buffer. The bound complexes were eluted by adding SDS Sample Buffer [62.5 mM Tris-HCl pH 6.8, 2% (v/v) SDS, 10% (v/v) glycerol, 0.01 (w/v) bromophenol blue], followed by SDS-PAGE and western blotting with antibodies against MIWI, MILI, TDRD1, or TDRD6.

### Histological analysis

For histology, testes were fixed overnight with 4% paraformaldehyde in PBS at 4°C, washed in PBS, embedded in paraffin, and sectioned at 5 μm. The sections were prepared and stained with hematoxylin and eosin.

### Bisulfite methylation analysis

Genomic DNA isolated from the testes of 8-week-old mice was treated with bisulfate using the Epitect Bisulfite Kit (Qiagen, Germantown, MD). A long terminal repeat (LTR) region of IAP on chromosome 3qD was arbitrarily selected for analysis by nested PCR. The 5’ region of the L1-type Gf was amplified by specific primers [5]. The products were cloned with the TOPO TA Cloning Kit (Invitrogen), and the resulting single colonies were picked and grown. Plasmid DNAs were extracted from the colonies, and the insert DNA of each plasmid was sequenced.

## Results and Discussion

### The two N-terminal CHHC-domain repeats of GTSF1L and GTSF2 are highly homologous

GTSF1L and GTSF2 are UPF0224 proteins, which contain two N-terminal copies of a CHHC-type Zn-finger domain (**Fig 1A and 1B**); this domain is predicted to recognize and bind RNA [19]. Amino-acid sequence alignment of mouse GTSF1, GTSF1L, and GTSF2 proteins (**Fig 1B**) showed that the N-terminal regions containing these Zn-finger domains were highly homologous in these three UPF0224-family genes, whereas the homology of the C-terminal regions, whose function is unknown, was low.

### *Gtsf1l* and *Gtsf2* are abundantly expressed in the testis

**Fig 2:**
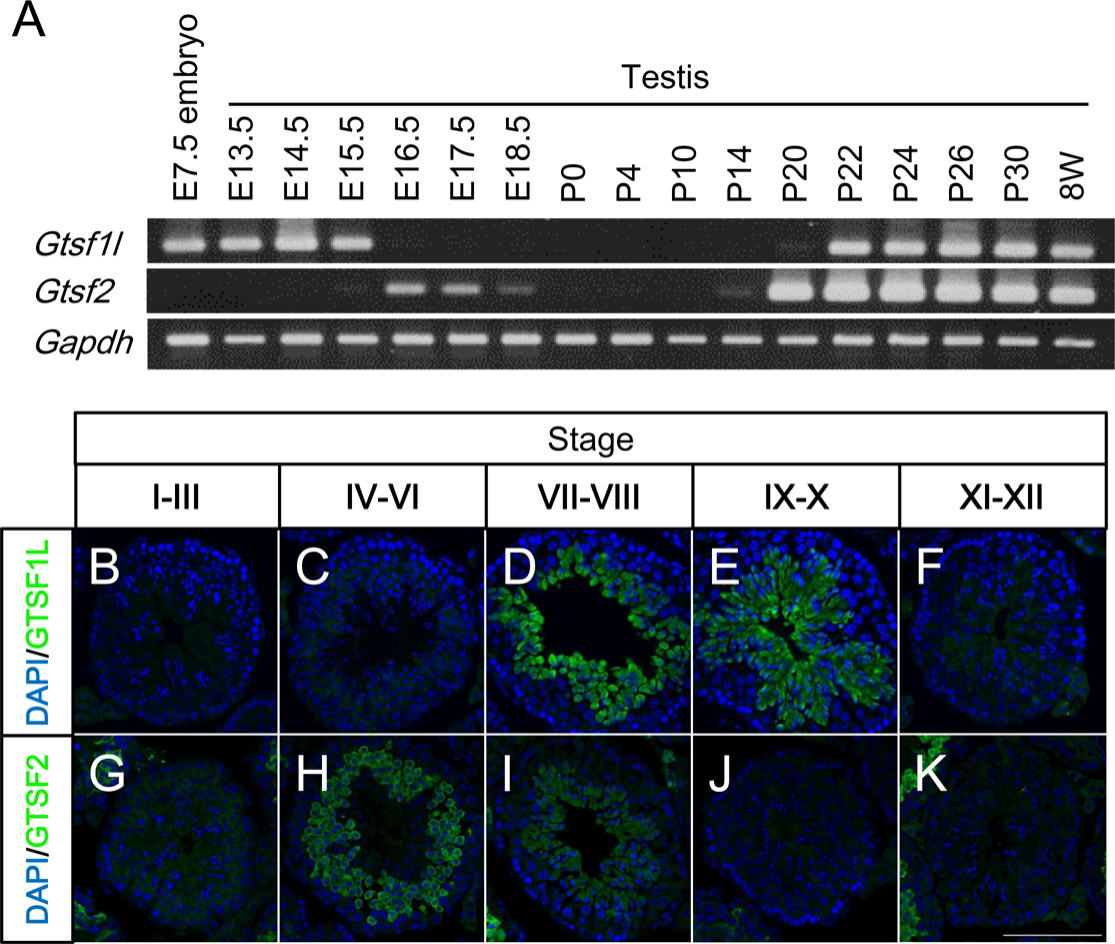
*Gtsf1l* and *Gtsf2* expression in male germ-cell development. (A) RT-PCR of *Gtsf1l*, *Gtsf2*, and *Gapdh* mRNAs using total RNA from the whole embryo at E7.5 and from the testes at various time points from E13.5 to 8 weeks. P: postnatal day. (B-K) Frozen sections of adult testis were immunostained with anti-GTSF1L and anti-GTSF2 antibodies (green), revealing GTSF1L (B-F) and GTSF2 (G-K) protein expression in the seminiferous tubules at developmental stages I-III (B, G), IV-VI (C, H), VII-VIII (D, I), IX-X (E, J), and XI-XII (F, K). Nuclei were stained with DAPI (blue). Scale bar: 100 μm.

We previously reported that *Gtsf1l* mRNA is detected in the mouse brain, lung, spleen, thymus, testis, ovary, and ES cells, and that *Gtsf2* mRNA is detected in unfertilized eggs and the testis [11]. Here, we analyzed the *Gtsf1l* and *Gtsf2* mRNA expression in embryonic and postnatal testes by RT-PCR (**Fig 2A**). *Gtsf1l* transcripts were detected earlier than embryonic day (E)15.5, and from postnatal day (P)22 onward. *Gtsf2* transcripts were detected from E16.5 to E18.5, and from P20 onward. Thus, the expression of both genes decreased temporarily around the time of birth. In the embryonic testes, *Gtsf1l* and *Gtsf2* were expressed sequentially and alternately. In the postnatal period, these genes began to be expressed at different time points: *Gtsf2* at P20 and *Gtsf1l* at P22. Because the first wave of spermatogenesis reaches the stage of round spermatids at P20 [20], these results suggest that the *Gtsf1l* and *Gtsf2* mRNA expression is initiated at the stage of round spermatids or later.

To determine the GTSF1L and GTSF2 expression patterns during spermatogenesis, we analyzed adult mouse testes by immunofluorescence using antibodies against the C-terminal portions of GTSF1L and GTSF2 (**Fig 1A**). We found distinct GTSF1L and GTSF2 signals in the inner space of the seminiferous tubules (**Fig 2B–2K**). Spermatogonia, spermatocytes, or round spermatids were not stained for GTSF1L (**Fig 2B–2F**). The cytoplasm of early elongating spermatids was strongly stained for GTSF1L at stage VII-X, but the staining decreased in the late elongating spermatids at stage XI-XII. On the other hand, the GTSF2 staining was not detected in spermatogonia, spermatocytes, early round spermatids, or late elongating spermatids, but was clearly seen in the cytoplasm of late round spermatids at stage IV-VI (**Fig 2G–2K**). GTSF2 staining decreased in the early elongating spermatids at stage VII-VIII. These results indicated that the GTSF2 and GTSF1L proteins are expressed sequentially and transiently at specific stages of spermatogenesis in the postnatal testes. Since proteins that are exclusively expressed in spermiogenic cells are rare, the expression patterns of GTSF2 and GTSF1L make them useful specific markers: GTSF2 for late round spermatids and GTSF1L for early elongating spermatids.

### GTSF1L and GTSF2 interact with the MIWI, MILI, and TDRD1 complexes

**Fig 3:**
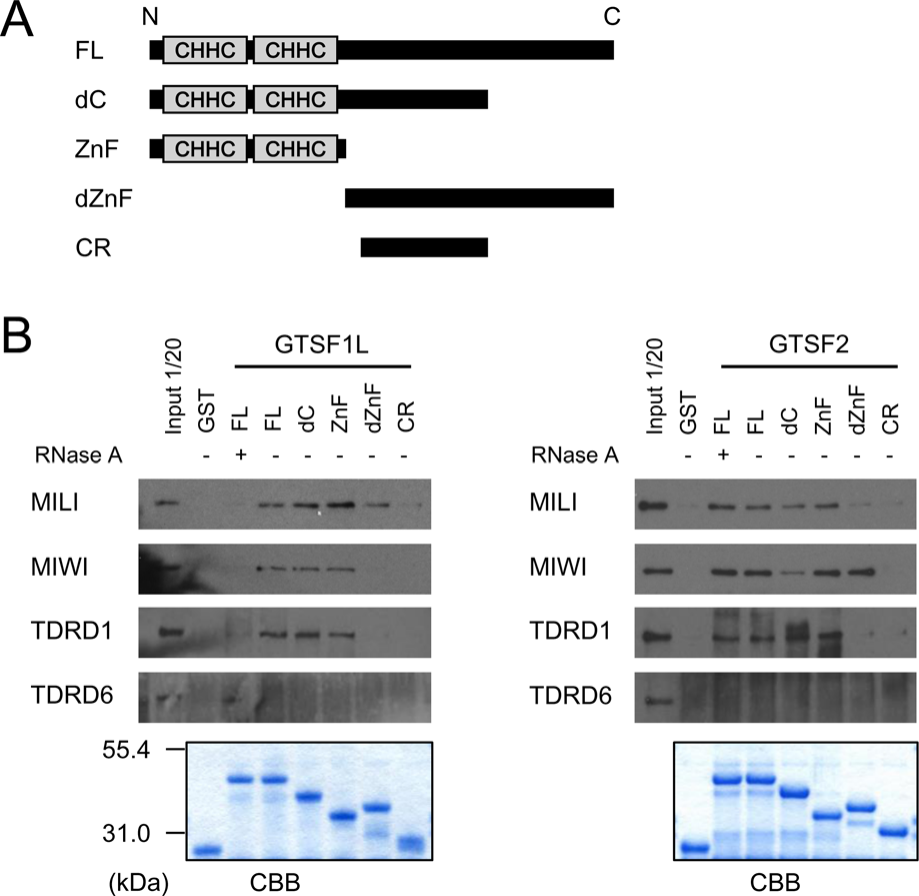
GTSF1L and GTSF2 interact with PIWI and Tudor complexes. (A) Diagram showing the GST-fused GTSF1L or GTSF2 protein with various deletions. FL: the full-length protein; dC: the protein with a C-terminal deletion; ZnF: the N-terminal region of the protein, which contains the Zn-finger domains; dZnF: the protein with an N-terminal deletion; CR: the central region. (B) Pull-down experiments using of GST-fused GTSF1L or GTSF2 or their deletion mutants. Pulled-down proteins were analyzed by western blotting with antibodies against MILI, MIWI, TDRD1, or TDRD6. Testis lysates were untreated (-) or pretreated (+) with RNase A. Part of each pull-down fraction was separated by SDS-PAGE and stained with Coomassie Brilliant Blue (CBB).

Recently, *Drosophila* Gtsf1 was reported to associate with PIWI proteins via its central region [21,22]. Furthermore, we found that mouse GTSF1 associates with the MIWI, MILI, and MIWI2 complexes via its central region (unpublished). The timing of expression of the MIWI, MILI, TDRD1, and TDRD6 complexes partially overlaps that of GTSF1L and GTSF2. Therefore, to determine whether GTSF1L and GTSF2 interact with the MIWI, MILI, TDRD1, or TDRD6 complex, we performed pull-down experiments from mouse testis lysates using recombinant GTSF1L and GTSF2 proteins fused to GST. We found that full-length GTSF1L and GTSF2 pulled down MIWI, MILI, or TDRD1, but not TDRD6 (**Fig 3B**). Further analysis showed that GTSF1L and GTSF2 associated with these complexes via an N-terminal Zn-finger domain, rather than through a central region like GTSF1. This difference in the interacting domain may suggest functional differences between GTSF1 and GTSF1L or GTSF2. To clarify whether this interaction depended on RNA, we performed pull-down assays using RNase A. We found that GTSF1L’s pull-down of the MIWI, MILI, and TDRD1 complexes depended on RNA (**Fig 3B, left panel**), but GTSF2’s pull-down was independent of RNA (**Fig 3B, right panel**).

### *Gtsf1l*^*-/*-^ and *Gtsf2*^*-/*-^ mice have normal spermatogenesis

**Fig 4:**
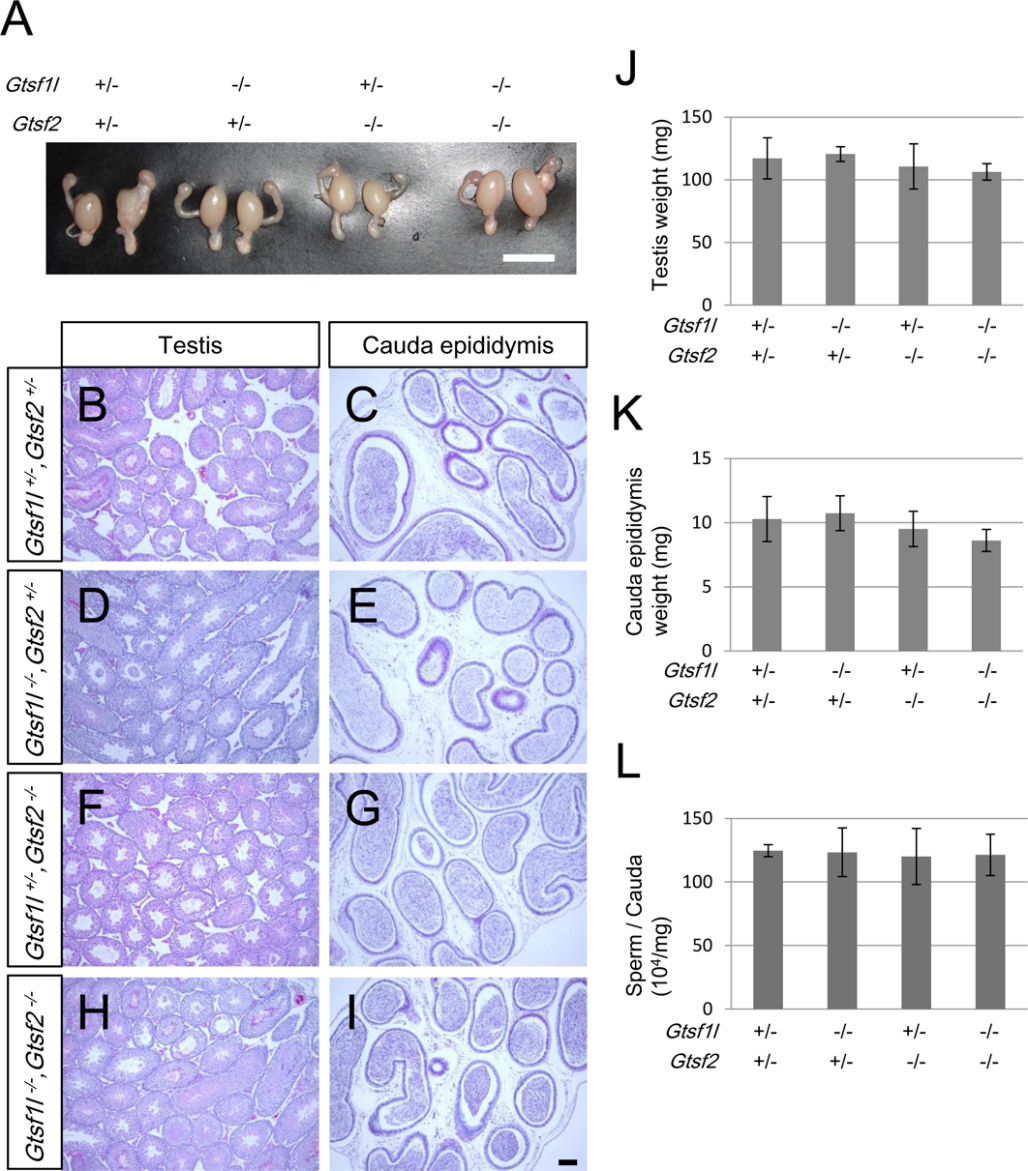
Normal spermatogenesis in mice lacking *Gtsf1l* and/or *Gtsf2*. (A) Representative images of testes from 8-week-old *Gtsf1l*^+/-^/*Gtsf2*^*+/*-^, *Gtsf1l*^*-/*-^*/Gtsf2*^*+/*-^, *Gtsf1l*^*+/*-^*/Gtsf2*^*-/*-^, and *Gtsf1l*^*-/*-^/*Gtsf2*^*-/*-^ mice. Scale bar: 1 mm. (B-I) Hematoxylin/eosin-stained sections of the testis (B, D, F, and H) and cauda epididymis (C, E, G, and I) from 8-week-old *Gtsf1l*^+/-^/*Gtsf2*^*+/*-^ (B, C), *Gtsf1l*^*-/*-^*/Gtsf2*^*+/*-^ (D, E), *Gtsf1l*^*+/*-^*/Gtsf2*^*-/*-^ (F, G), and *Gtsf1l*^*-/*-^/*Gtsf2*^*-/*-^ (H, I) mice. Scale bar: 200 μm. (J, K) Mean weight of the testis (J) and cauda epididymis (K) in 8-week-old mice. Error bars represent standard deviation. (L) Total sperm counts per mg cauda epididymis in 8-week-old mice. Error bars represent standard deviation.

A lack of MIWI, MILI, or TDRD1 is reported to cause infertility [23–25]. A defect in the piRNA pathway is also thought to cause infertility [3,5,8]. *Gtsf1*-null mice are infertile, and derepressed retrotransposons are observed in their testes [12]. To reveal the *in vivo* functions of *Gtsf1l* and *Gtsf2*, we generated *Gtsf1l*- and *Gtsf2*-knockout mice. We used the CRISPR/Cas9 system to obtain a frame-shifted *Gtsf1l* allele (**S1 Fig**). We also generated mice with a floxed *Gtsf2* allele by inserting a loxP sequence into intron 1 and intron 4 of *Gfsf2* in ES cells (**S2A Fig**). To generate a *Gtsf2*-null allele, the *Gtsf2*-flox mice were crossed with CAG-Cre mice, deleting the region from exon 2 to 4. We confirmed the lack of *Gtsf1l* and *Gtsf2* mRNAs and their products in the mutant mice by RT-PCR, western blotting, and immunostaining (**S3A–S3F Fig**). Both male and female *Gtsf1l*- and *Gtsf2*-null mice were fertile. *Gtsf1l*/*Gtsf2* double-null mice were also fertile. The weight of the testes and cauda epididymes in the *Gtsf1l*^*-/*-^*/Gtsf2*^*+/*-^, *Gtsf1l*^*+/*-^/*Gtsf2*^*-/*-^, and *Gtsf1l*^*-/*-^/*Gtsf2*^*-/*-^ mice was comparable to that in *Gtsf1l*^+/-^/*Gtsf2*^*+/*-^ mice (**Fig 4A**). Histologically, the testes and cauda epididymes in these mutants appeared normal (**Fig 4B–4I**). The sperm counts were also comparable among these four genotypes (**Fig 4J–4L**). These results suggested that *Gtsf1l* and *Gtsf2* are dispensable for spermatogenesis in mice.

Next, to reveal whether *Gtsf1l* and/or *Gtsf2* are involved in suppressing retrotransposons, we examined the retrotransposon expression and CpG DNA methylation in the retrotransposon promoter regions. First, we examined the methylation by bisulfate sequencing analysis (**S4A Fig**). Next, we examined the retrotransposon expression by RT-qPCR and immunofluorescence (**S4B–S4F Fig**). Our results indicated that the loss of *Gtsf1l* and/or *Gtsf2* does not affect retrotransposon suppression.

This study found that although GTSF1L and GTSF2 associate with the PIWI and Tudor complexes, *Gtsf1l* and *Gtsf2* are dispensable for spermatogenesis and retrotransposon suppression. Considering the specific expression patterns of *Gtsf1l* and *Gtsf2* and the structure of their products, these genes may play some roles in spermatogenesis, but may be required only in specific circumstances or genetic backgrounds. The homology between GTSF1L and GTSF2 is high (approximately 78%) in the N-terminal regions (ZnF; **Fig 3A**), but low (approximately 30%) in the C-terminal regions (dZnF; **Fig 3A**). These C-terminal regions may contribute to the difference of GTSF1L and GTSF2 in the affinity to other molecules and/or the functions. Examining the effects of *Gtsf1l* and/or *Gtsf2* overexpression may further clarify the roles of GTSF1L and GTSF2 in spermatogenesis in mice.

## Supporting Information

S1 Fig Targeting strategy for the *Gtsf1l* gene locus. (A) *Gtsf1l* exon 2 sequences: sequences used for genotyping primers are underlined, and ORF sequences are in bold letters. The PAM and CRISPR target sequences are shown in red and green, respectively. (B) ORF sequences of the mutated allele in *Gtsf1l*. Red and green letters show inserted sequences speculated to derive from the tRNA-histidine guanylyltransferase 1-like (*Thg1l*) gene on chromosome 11 (red) and the predicted gene 2420 (*Gm2420*) pseudogene on chromosome 5 (green). Blue letters indicate a postulated stop codon in the mutated allele. Postulated ORF sequence in the mutated allele is underlined.
(TIF)

S2 Fig Targeting strategy for the *Gtsf2* gene locus. (A) Gene-targeting strategy for *Gtsf2*, showing exons (black bars), LoxP and FRT sites (black and red arrowheads, respectively), and start (*) and stop (@) codons. (B) Southern blot of genomic DNA derived from wild-type and heterozygous ES cells. The wild-type allele generates a 7.0-kb fragment, while the targeted allele generates a 4.5-kb fragment. (C) RT-PCR analysis of *Gtsf2* mRNA in the testes from 8-week-old mice, with *Gapdh* mRNA as the loading control. (D) Western blot of the lysate from BMT-10 cells transfected with pGTSF2 and from the testes of *Gtsf2*^*+/*-^ and *Gtsf2*^*-/*-^ mice, using anti-GTSF2 and β-ACTIN antibodies.
(TIF)

S3 Fig Analysis of mRNA and protein in *Gtsf1l* and *Gtsf2* mutant mice. (A) RT-PCR analysis of the *Gtsf1l* and *Gtsf2* mRNAs in the testes from 8-week-old mice. *Gapdh* mRNA served as the loading control. (B) Western blots of testis lysates from 8-week-old *Gtsf1l*^+/+^/*Gtsf2*^*+/+*^, *Gtsf1l*^*-/*-^*/Gtsf2*^*+/+*^, *Gtsf1l*^*+/+*^*/Gtsf2*^*-/*-^, and *Gtsf1l*^*-/*-^/*Gtsf2*^*-/*-^ mice using anti-GTSF1L, anti-GTSF2, and β-ACTIN antibodies. (C-F) Frozen sections of adult testis from 8-week-old *Gtsf1l*^+/+^/*Gtsf2*^*+/+*^ (C, E) and *Gtsf1l*^*-/*-^/*Gtsf2*^*-/*-^ (D, F) mice were immunostained with anti-GTSF1L (C,D) and anti-GTSF2 (E,F) antibodies (green). Nuclei were stained with DAPI (blue). Scale bars: 200 μm.
(TIF)

S4 Fig Expression and regulatory-region methylation of retrotransposons in the testes from *Gtsf1l* and *Gtsf2* mutant mice. (A) Bisulfite sequencing analysis of Line-1 and IAP. The 5’-noncoding regions of type Gf Line-1 (GenBank accession No. D84391) and the 5.4-kb IΔ1-type IAP in chromosome 3qD were arbitrarily analyzed using previously described PCR primers [5]. Filled and open circles represent methylated and unmethylated CpGs, respectively; the percentages of methylated CpGs are shown. (B) Quantitative RT-PCR analysis of Line-1 and IAP expression in the testes from 8-week-old *Gtsf1l*^+/-^/*Gtsf2*^*+/*-^, *Gtsf1l*^*-/*-^*/Gtsf2*^*+/*-^, *Gtsf1l*^*+/*-^*/Gtsf2*^*-/*-^, and *Gtsf1l*^*-/*-^/*Gtsf2*^*-/*-^ mice; data were normalized to the *Gapdh* expression. Error bars indicate standard deviation. (C-F) Frozen sections of the adult testes from 8-week-old *Gtsf1l*^+/+^/*Gtsf2*^*+/+*^ (C, E) and *Gtsf1l*^*-/*-^/*Gtsf2*^*-/*-^ (D, F) mice were immunostained using anti-L1ORF1p (C, D) and anti-IAP GAG (E, F) antibodies (green). Nuclei were stained with DAPI (blue). Scale bar: 500 μm.
(TIF)
