# Supplementary figures and images for: Gtsf1l and Gtsf2 Are Specifically Expressed in Gonocytes and Spermatids but Are Not Essential for Spermatogenesis
Source: PLoS One. 2016 Mar 1;11(3):e0150390. doi: 10.1371/journal.pone.0150390 (PMC4773171; doi:10.1371/journal.pone.0150390)

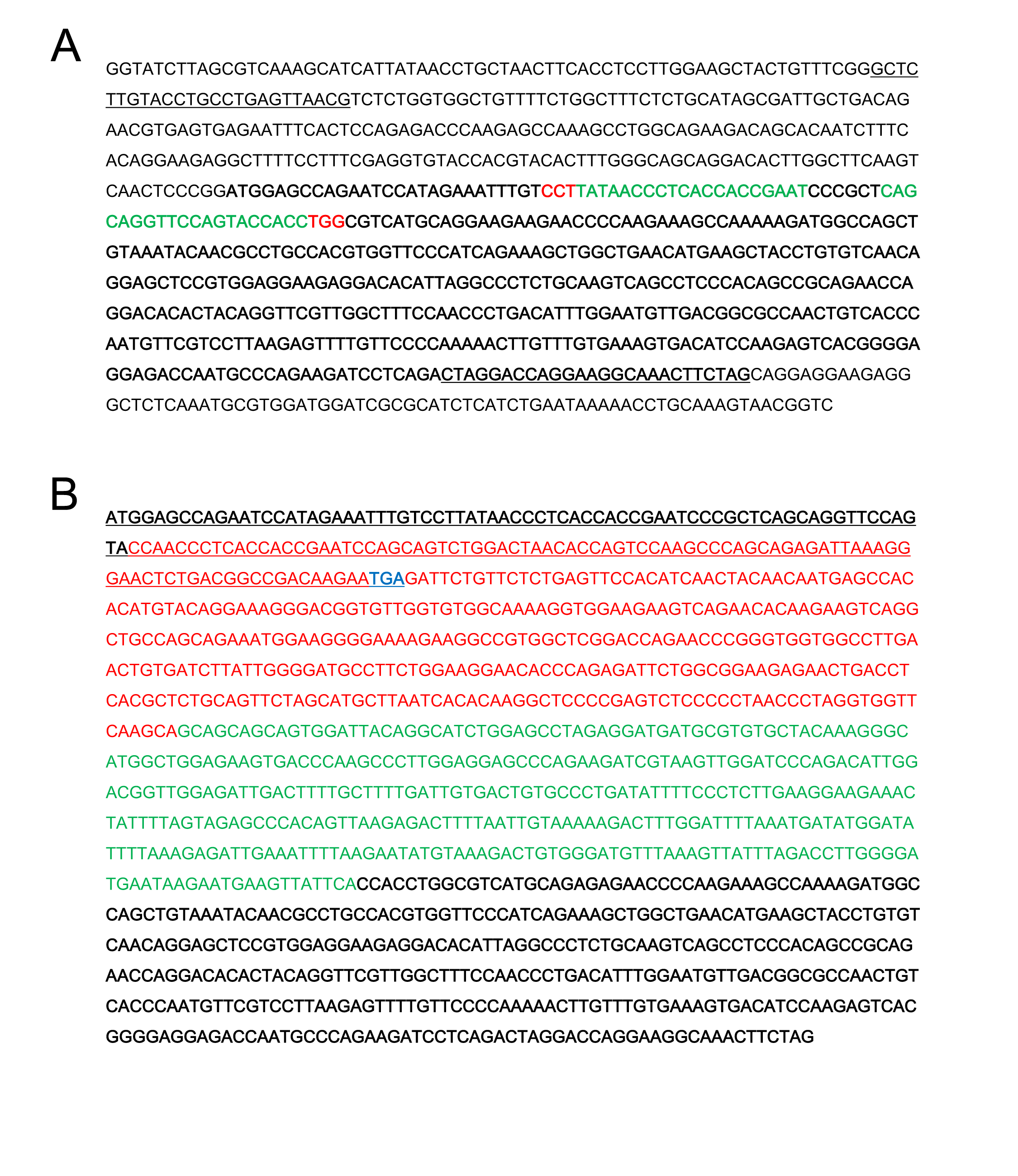

Supplement: S1 Fig — (A) Gtsf1l exon 2 sequences: sequences used for genotyping primers are underlined, and ORF sequences are in bold letters. The PAM and CRISPR target sequences are shown in red and green, respectively. (B) ORF sequences of the mutated allele in Gtsf1l. Red and green letters show inserted sequences speculated to derive from the tRNA-histidine guanylyltransferase 1-like (Thg1l) gene on chromosome 11 (red) and the predicted gene 2420 (Gm2420) pseudogene on chromosome 5 (green). Blue letters indicate a postulated stop codon in the mutated allele. Postulated ORF sequence in the mutated allele is underlined. (TIF) [file pone.0150390.s001.tif]

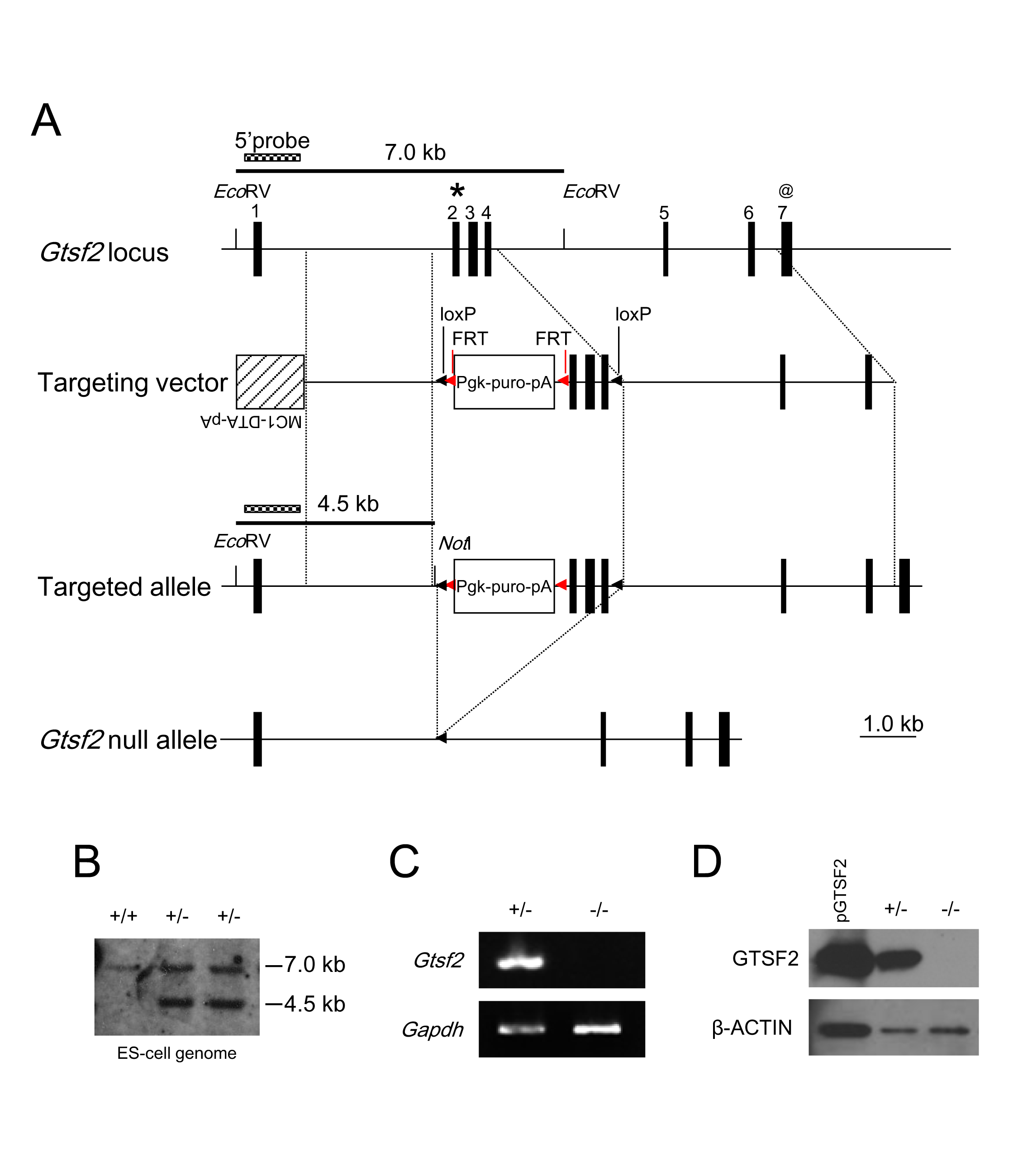

Supplement: S2 Fig — (A) Gene-targeting strategy for Gtsf2, showing exons (black bars), LoxP and FRT sites (black and red arrowheads, respectively), and start (*) and stop (@) codons. (B) Southern blot of genomic DNA derived from wild-type and heterozygous ES cells. The wild-type allele generates a 7.0-kb fragment, while the targeted allele generates a 4.5-kb fragment. (C) RT-PCR analysis of Gtsf2 mRNA in the testes from 8-week-old mice, with Gapdh mRNA as the loading control. (D) Western blot of the lysate from BMT-10 cells transfected with pGTSF2 and from the testes of Gtsf2+/- and Gtsf2-/- mice, using anti-GTSF2 and β-ACTIN antibodies. (TIF) [file pone.0150390.s002.tif]

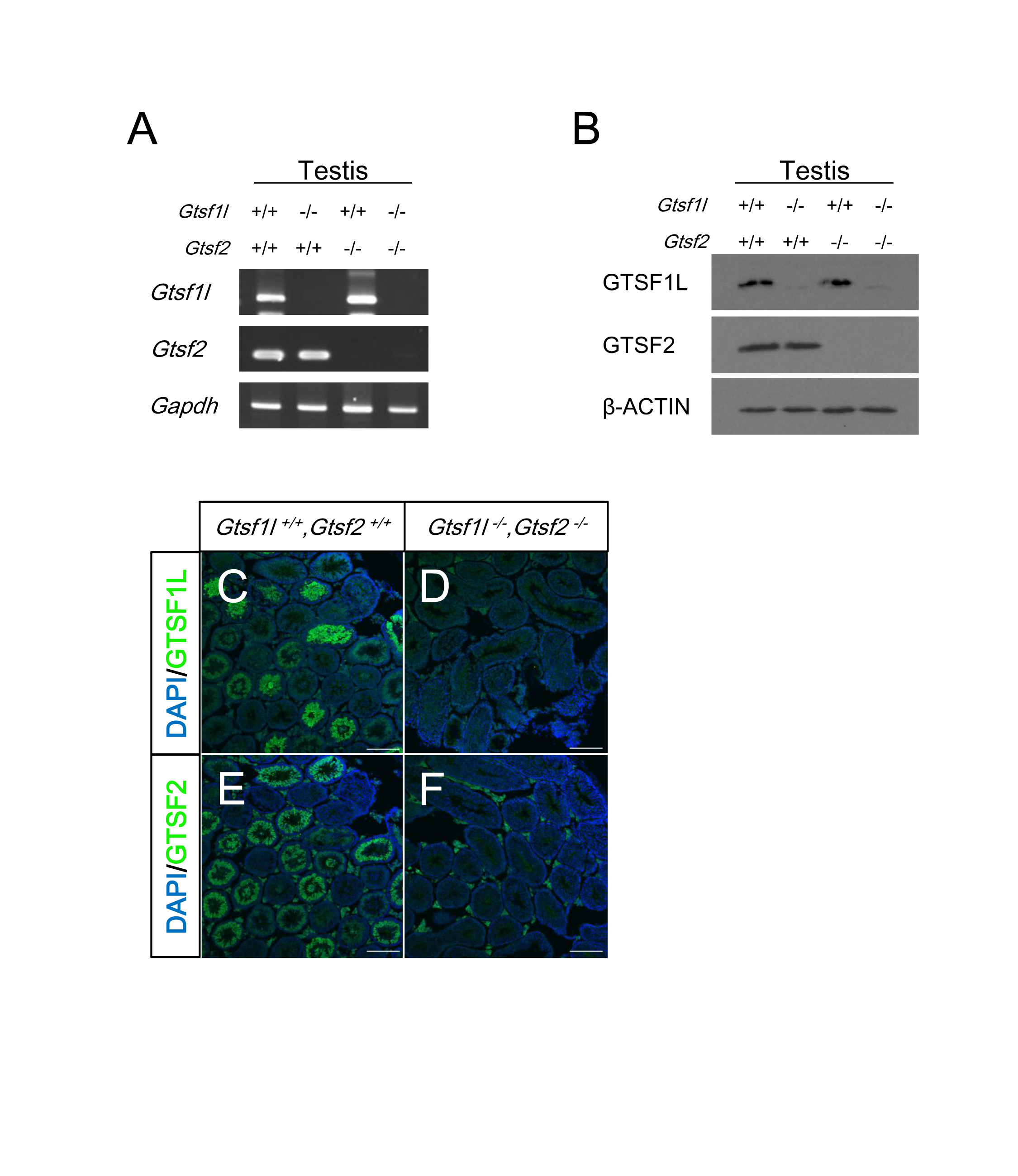

Supplement: S3 Fig — (A) RT-PCR analysis of the Gtsf1l and Gtsf2 mRNAs in the testes from 8-week-old mice. Gapdh mRNA served as the loading control. (B) Western blots of testis lysates from 8-week-old Gtsf1l+/+/Gtsf2+/+, Gtsf1l-/-/Gtsf2+/+, Gtsf1l+/+/Gtsf2-/-, and Gtsf1l-/-/Gtsf2-/- mice using anti-GTSF1L, anti-GTSF2, and β-ACTIN antibodies. (C-F) Frozen sections of adult testis from 8-week-old Gtsf1l+/+/Gtsf2+/+ (C, E) and Gtsf1l-/-/Gtsf2-/- (D, F) mice were immunostained with anti-GTSF1L (C,D) and anti-GTSF2 (E,F) antibodies (green). Nuclei were stained with DAPI (blue). Scale bars: 200 μm. (TIF) [file pone.0150390.s003.tif]

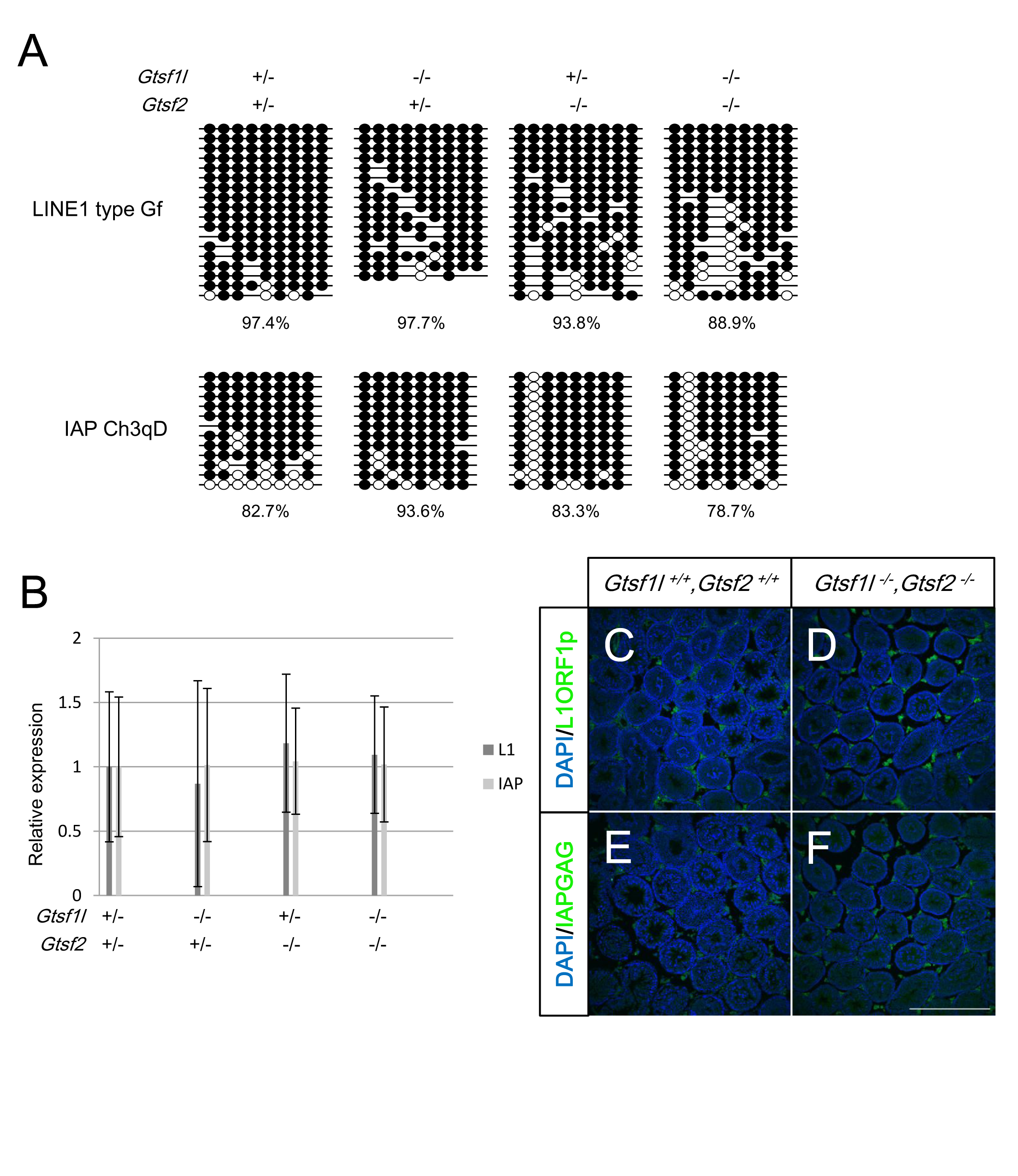

Supplement: S4 Fig — (A) Bisulfite sequencing analysis of Line-1 and IAP. The 5’-noncoding regions of type Gf Line-1 (GenBank accession No. D84391) and the 5.4-kb IΔ1-type IAP in chromosome 3qD were arbitrarily analyzed using previously described PCR primers [5]. Filled and open circles represent methylated and unmethylated CpGs, respectively; the percentages of methylated CpGs are shown. (B) Quantitative RT-PCR analysis of Line-1 and IAP expression in the testes from 8-week-old Gtsf1l+/-/Gtsf2+/-, Gtsf1l-/-/Gtsf2+/-, Gtsf1l+/-/Gtsf2-/-, and Gtsf1l-/-/Gtsf2-/- mice; data were normalized to the Gapdh expression. Error bars indicate standard deviation. (C-F) Frozen sections of the adult testes from 8-week-old Gtsf1l+/+/Gtsf2+/+ (C, E) and Gtsf1l-/-/Gtsf2-/- (D, F) mice were immunostained using anti-L1ORF1p (C, D) and anti-IAP GAG (E, F) antibodies (green). Nuclei were stained with DAPI (blue). Scale bar: 500 μm. (TIF) [file pone.0150390.s004.tif]
